# Polyamine Induction of Secondary Metabolite Biosynthetic Genes in Fungi Is Mediated by Global Regulator LaeA and α-NAC Transcriptional Coactivator: Connection to Epigenetic Modification of Histones

**DOI:** 10.3390/molecules30193903

**Published:** 2025-09-27

**Authors:** Juan F. Martín

**Affiliations:** Departamento de Biología Molecular, Área de Microbiología, Universidad de León, 24071 León, Spain; jf.martin@unileon.es

**Keywords:** polyamines, spermidine, 1,3-diaminopropane, LaeA, α-NAC, epigenetics, histones, secondary metabolites, fungi

## Abstract

Polyamines are polycationic compounds present in all living cells that exert functions at different levels in the metabolism. They bind to DNA and RNA and modulate DNA replication and gene expression. Some of these regulatory effects are exerted by promoting condensation of nucleosomes, a mechanism closely connected with epigenetic modification by histone methylation and acetylation. The polyamines 1,3-diaminopropane and spermidine induce expression of the global regulator LaeA and increase by several folds the formation of the α-NAC transcriptional co-activator, a subunit of the nascent polypeptide-associated complex. The global regulator LaeA controls the switch from primary growth to secondary metabolite production and differentiation when an essential nutrient in the growth medium becomes limiting. α-NAC exerts significant control over the biosynthesis of secondary metabolites and fungal pathogenicity on plants. When purified α-NAC protein is added to a tomato host plant, it induces plant resistance to fungal infections and triggers the development of system-acquired resistance in other plants. Spermidine extends the life of yeast cells and prolongs the half-life of penicillin gene transcripts in *Penicillium chrysogenum*. This article discusses advances in the basis of understanding the mechanism of plant–fungi interaction and the effect of small fungal metabolites and epigenetic modifiers in this interaction.

## 1. Introduction

Filamentous fungi produce a great variety of secondary metabolites (also named specialised metabolites, SMs) that play important roles in the pharmaceutical industry, e.g., as antibiotics, antitumour agents, immunosuppressants, anticholesterolemic products, and enzyme inhibitors, among others [1,2].

During recent decades, numerous SMs with diverse biological and pharmacological activities have been isolated, but there is a consensus that the number of SMs encoded in filamentous fungi is underestimated. Following genome sequencing of diverse fungi, an immense number of genes encoding polyketide synthases (PKSs), non-ribosomal peptide synthetases (NRPSs), hybrid multifunctional PKSs–NRPSs, and terpenoid cyclases have been documented [3]; however, most of these genetically characterized gene clusters are silent or very poorly expressed [4,5,6,7].

Increasing accumulated evidence indicates that some nutritional factors control the biosynthesis of SMs at the transcriptional and translational levels. Among these important factors are polyamines, which regulate the growth, differentiation, pathogenicity, and formation of secondary metabolites in fungi. In this article, we review recent advances and the present status of the molecular mechanisms that control the biosynthesis of SMs by polyamines.

Polyamines are intracellular organic polycations; the four ornithine-derived polyamines are the diamines putrescine (1,4-diaminobutane) and 1,3-diaminopropane (DAP), the triamine spermidine, and the tetraamine spermine, all of which occur in most filamentous fungi.

Polyamines are essential for the growth of animal and fungal cells; indeed, polyamine auxotroph mutants of *Saccharomyces cerevisiae* that require spermidine for growth were isolated [8]. This is due to the fact that spermidine is the direct precursor of the rare amino acid hypusine, which plays a critical role in the modification of the translation/initiation factor eIF-5A that is essential for protein synthesis [9]. The intracellular concentration of polyamines is strictly regulated by de novo biosynthesis, degradation, secretion, and uptake from extracellular polyamine sources. This results in an optimal level of diamines for the different stages of growth of the cell cycle [10].

### Historical Overview of the Studies of the Role of Polyamines in Animal and Fungal Cells

The role of polyamines in animal cells has received great attention because of their involvement in the control of tumour cell growth [11,12,13], but knowledge of the molecular mechanisms of polyamines’ effect in filamentous fungi has advanced slowly. Due to their polycationic character, polyamines interact strongly with the negative charge of DNA and RNA, stabilizing their structures, particularly in the case of mRNA, and also interact with some specific proteins, stabilizing them.

The DNA and proteins in chromatin are organized as arrays of nucleosomes. Each nucleosome core is an octamer of histone H2A-H2B-H3-H4 (two copies of each) wrapped by a DNA stretch of about 165 base pairs. Early studies showed that polyamines alter the configuration and increase the stability of nucleosome core particles [14]. They also facilitate oligomerization of nucleosomes in vitro, resulting in the condensation of chromatin, an effect that is inhibited by hyperacetylation of histones [15]. It was found that high levels of polyamines stimulate histone acetyltransferase (HAT) activity, resulting in chromatin hyperacetylation [16,17,18]. Natural polyamines and some chemical analogues have the capability to bind nucleosomes and form condensed chromatin. This finding led to the proposal that the effect of polyamines is related to chromatin modifications [11]. In the last decade, there has been great interest in understanding the role of chromatin modifications in the expression of genes of secondary metabolite gene clusters.

As will be shown in detail below, increasing evidence suggests that polyamines, particularly spermidine and 1,3-diaminopropane (DAP), control the expression of SMs in different filamentous fungi through an effect on histone modifications, including acetylation/deacetylation, methylation/demethylation, phosphorylation, ubiquitination, and sumoylation, and this affects the expression of SM biosynthetic genes [17,19,20]. Histone methylations take place in basic amino acids, particularly at the lysine or arginine located at the histone tail [17]. The six-carbon skeleton of lysine has an adequate length and position of the C6 amino group in the histone tail to be recognized by the histone methyl transferases that use S-adenosylmethionine (SAM) as a methyl donor. Methylations by histone methyl transferases may introduce one to three methyl groups at lysine residues, resulting in mono-, di-, and three-methylated lysine. When SAM is limiting, the methylated product is normally the monomethylated histone [21]. However, highly methylated histones are demethylated by histone dimethyl transferases and the methylation/demethylation balance is important in the final degree of histone modification [22,23,24]. Histone acetyl transferases acetylate lysine residues in the histone tails and promote the formation of open chromatin that facilitates gene expression. Histone deacetyltransferases (HDACs) remove the acetyl groups, favouring the condensation of chromatin [25]. In summary, natural polyamines and some polyamine analogues interact with the DNA structure and chromatin modifying enzymes such as lysine-specific histone demethylases (DMS1), histone acetyltransferases, and histone deacetylases (HDACs) [20] controlling the chromatin condensed or relaxed structure. The condensed chromatin structure is transcriptionally inactive (heterochromatin), while a relaxed chromatin structure (euchromatin) favours transcription of the encoded genes [25,26]. Importantly, in fungi, polyamine regulation is associated with the global regulator LaeA and modification by chromatin-modifying enzymes.

**Figure 1:**
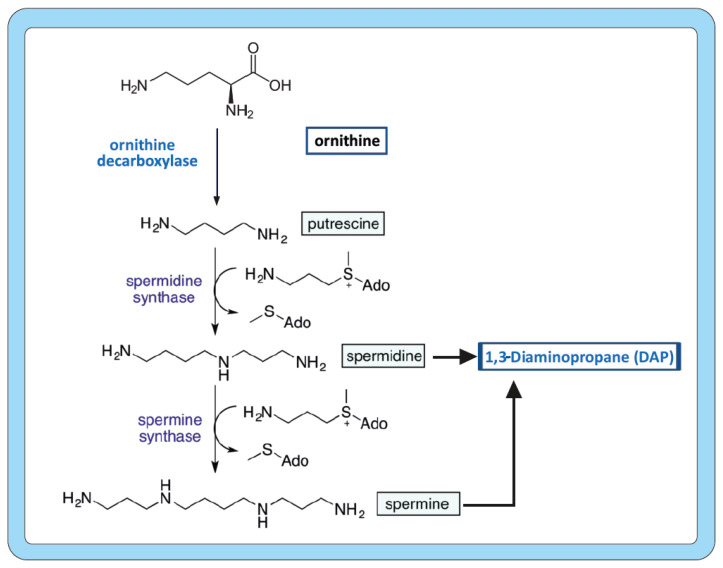
Biosynthetic pathway of the major polyamines. Note that spermidine and spermine break down, producing 1,3-diaminopropane. The five-carbon amino acid ornithine is synthesized as an intermediate of the arginine pathway and may also be formed by catabolism of arginine via agmatine.

Polyamines control the expression of some genes in eukaryotic cells at the transcriptional level [10,27,28]; one of the major points of action of polyamines is the transcriptional control of SM biosynthesis at two levels, (1) expression of the global regulator *laeA* gene [29] and (2) expression of the co-activator α-NAC (for nascent polypeptide-associated complex) protein that plays a key role in the interaction with and recruitment of additional transcriptional factors. Moreover, polyamines control several pathways in different animal cells and filamentous fungi exerted at the translational or posttranslational level; these changes include hypusine modification of the translation-initiation factor eIF-5A [9,10,30]. Another important recent finding is that polyamines regulate the folding of the 5’untranslated region (UTR) of the mRNA of S-adenosylmethionine decarboxylase, one of the genes that play a crucial role in polyamine metabolism (Figure 1) [31,32]. Studies at the translational level have been reviewed by other authors [10] and are not detailed here; this article is focused on polyamine control of fungal metabolism at the transcriptional level.

## 2. Biosynthesis of Polyamines

Polyamines derive from the five-carbon amino acid ornithine and, in a few cases, from arginine via agmatine by the action of agmatinase. Putrescine is formed by the decarboxylation of ornithine and is converted to spermidine by condensing with a propylamine unit; an additional propylamine moiety converts spermidine to spermine (Figure 1). Both propylamine units derive from S-adenosylmethionine (SAM) by the combined action of the following two enzymes: first, SAM is decarboxylated to decarboxyl-SAM (dcSAM, also named dc-Adomet) by the action of the SAM decarboxylase, and then the three-carbon propylamine fragment is transferred from dcSAM to putrescine by the spermidine synthase. The SAM decarboxylase, encoded by a gene without introns, plays a key role in the splitting of SAM, exposing the three-carbon unit to the action of the following enzyme of the pathway, spermidine synthase [30,31,32], in a very complex mechanism that has received considerable attention. The SAM decarboxylases contain several conserved characteristic sequences that include sites for autocleavage and for interaction with putrescine. This indicates that the SAM decarboxylase processing mechanism is very well conserved in different living cells.

The major diamines are putrescine, spermidine, and spermine, which occur in almost all filamentous fungi in the intracellular range of 1 to 10 mM, whereas 1,3-diaminopropane is considered a rare diamine that may not be present in all filamentous fungi [32].

## 3. Role of Polyamines in the Biosynthesis of Fungal Secondary Metabolites

Transcriptional regulation and histone modifications are key mechanisms in the control of secondary metabolite gene expression. Polyamines regulate the expression of many secondary metabolites that belong to different classes of natural products.

### 3.1. Induction of Penicillin Gene Expression by Polyamines

*P. chrysogenum* lacks a regulatory gene located within the penicillin cluster [33,34,35], and the mechanisms that control the signal cascade involved in penicillin biosynthesis are poorly understood [35,36].

A penicillin biosynthesis autoinducer molecule has been isolated from filtered broth of *P. chrysogenum* and *Acremonium chrysogenum* stationary growth-phase cultures. Mass spectrometry and NMR chemical analysis of this compound indicate that the autoinducer is 1,3-diaminopropane (DAP) [37]. This compound is a rare three-carbon diamine which is part of the structure of the triamine spermidine, a precursor of pantothenic acid and coenzyme A (CoA), which favour the formation of β-lactam antibiotics [32].

DAP and spermidine also have regulatory functions; these polyamines, separately, showed the same response of protein changes in proteomic studies, suggesting that these two inducers are interconverted in vivo, as expected from polyamine biosynthesis studies [30,32]. Proteomic studies of cells supplemented with the inducers DAP or spermidine showed that there is an overrepresentation of enzymes involved in the biosynthesis of penicillin [38], as expected from the high level of transcription of the encoding genes, and particularly of the isopenicillin acyl transferase (IAT), as confirmed by immunoblotting studies [29]. The results of proteomic analysis are particularly interesting regarding the levels of two proteins, a polyamine oxidase and an aldehyde dehydrogenase, whose homologue proteins in *S. cerevisiae* are involved in β-alanine biosynthesis, a precursor of CoA and pantothenic acid. These last compounds are required for the modification of proteins by phosphopantetheinylation catalyzed by phosphopantetheinyl transferases [39] that activate NRPSs, PKSs, and fatty acid synthases [32]. The inducer effect exerted by DAP and spermidine is not observed in cultures supplemented with 1,4-diaminobutane (putrescine) or by the tetraamine spermine, suggesting that this effect is not a broad response to polyamines, which exert functions at a general level in all cells.

### 3.2. Polyamines Trigger the Biosynthesis of Trichothecenes, Aflatoxins, Lovastatin, Cephalosporin, and Other Secondary Metabolites in Filamentous Fungi

The discovery of DAP and spermidine as inducers of penicillin biosynthesis has generated interest in elucidating if these compounds exert a similar function in other fungi. Early evidence indicated that the fungal attack of some plants results in the stimulation of virulence of the infecting fungus, possibly by polyamine-mediated mechanisms. *Fusarium graminearum*, the wheat head blight disease agent, produces trichothecenes, e.g., deoxynivalenol (DON), which are very toxic for animals. Gardiner et al. [40] observed that exogenous polyamines trigger the synthesis of trichothecenes, including DON, and proposed that after infection, the fungus may provide polyamines. The polyamines’ control of the biosynthesis of trichothecenes is exerted at the transcriptional level, as shown in the expression of the TRI5 and TRI6 trichothecene biosynthetic genes [41].

*Aspergillus flavus* is an opportunist, saprophytic fungus that produces aflatoxins and is a pathogen of plants and seeds, including maize, peanuts, and groundnuts, among others. The aflatoxins present in plants or seeds infected by the fungus are toxic for animals. Majundar et al. [42] studied in *A. flavus* the effect of spermidine on *aflA* and *aflR* gene expression and aflatoxins biosynthesis, and demonstrated that spermidine produces a strong effect on growth, conidiation, and other SMs’ formation.

Another well documented example of regulation by DAP and spermidine of SM biosynthesis is that of the anticholesterolemic compound lovastatin produced by *Aspergillus terreus* [43]. Lovastatin is used as intermediate product for the chemical synthesis of semisynthetic statins, e.g., simvastatin. Addition of DAP or spermidine to the wild-type strain *A. terreus* resulted in an increasing yield of lovastatin (15–20%) with respect to the un-supplemented control culture, and simultaneously, expression of the biosynthetic genes *lovA* to *lovG* increased 1.2- to 3-fold. This enhanced expression was not due to increased transcription of polyamine biosynthetic genes, indicating that the stimulatory effect was due to a higher pool resulting from the externally added polyamines, but not from their endogenous formation. The polyamines’ effect was stronger in a genetically improved strain, *A. terreus* HY, that produced more than 200-fold higher lovastatin as compared to the wild-type strain. When supplemented with DAP or spermidine, *A. terreus* HY showed an increasing lovastatin yield of 30 to 45% [44,45]. However, the stimulatory effect of polyamines on high-producing strains is frequently strain-dependent due to their different genetic backgrounds, e.g., the HY mutant contains two copies of the *lovE* gene [44]. LovE is a cluster-situated zinc_2_-cys_6_ transcriptional regulator [46,47] that positively regulates the formation of lovastatin. The lovastatin transcriptional regulator LovE binds a specific sequence present upstream of most of the lovastatin genes, although informatic analysis reveals that this sequence is not present upstream of the transporter gene *lovT* and *lovE* itself [48].

An additional interesting example of the role of polyamines in the biosynthesis of SMs is related to the production of cephalosporin C (CPC) in *A. chrysogenum*. CPC yield increases about 17% and 21% in the presence of DAP and spermidine, respectively [48]. All the genes in the cephalosporin pathway (*pcbAB*, *pcbC cefD1*, *cefD2*, *cefEF*, and *cefG*) were upregulated by addition of DAP or spermidine to submerged cultures of wild-type *A. chrysogenum*. The enhancement of *cefG* expression in the supplemented cultures is important, because many of the *A. chrysogenum* strains accumulate the intermediate deacetyl-cephalosporin C (DAC) due to inefficient conversion by the DAC acetyl transferase of the DAC intermediate to the final product, cephalosporin C [49]. In most cases, the effect exerted by the spermidine was slightly higher than that observed upon addition of DAP. No increase was observed in the expression of the transporter gene *cefT* or in the peroxisome transporter genes *cefM* and *cefP* upon addition of the polyamine inducers [48]. The distinct effect of DAP and spermidine on the last steps of the CPC biosynthetic pathway is intriguing, and it has been proposed that the addition of spermidine does not support the conversion of DAC to cephalosporin due to a defect in acetyl-CoA, since large amounts of acetyl-CoA are required for the conversion of DAC into CPC [48]. This hypothesis needs further confirmation, but it is in agreement with the known effect of these diamines on the increase in the biosynthesis of CoA in *P. chrysogenum* [32,38]. Russian investigators, using random mutagenesis and selection of clones resistant to putrescine analogues, which are inhibitors of the ornithine decarboxylase, to avoid the feedback effect on this enzyme (Figure 1), developed *A. chrysogenum* HY, a strain producing above 200-fold cephalosporin C higher than the wild-type strain [30,32]. This strain contains higher internal levels of spermidine (5.2-fold) and spermine (4.5-fold) as compared to the wild-type strain *A. chrysogenum*.

The impressive high cephalosporin production of the genetically modified *A. chrysogenum* HY strain indicates that the strategy of removing the feedback regulation of the polyamine pathway by resistance to putrescine analogues is an interesting approach to increase the production of polyamine-regulated secondary metabolites.

In summary, the available information indicates that DAP and spermidine control expression of the biosynthesis of different classes of secondary metabolites, including β-lactams penicillin and cephalosporin C, polyketide-derived lovastatin, and isoprenoid-related trichothecenes. Probably this will be the case in the biosynthesis of many other SMs not yet studied.

## 4. Spermidine Increases Yeast Lifespan and Prolongs the Lifetime of Penicillin Gene Transcripts in *P. chrysogenum*

An important point in the biosynthesis of fungal SMs is the lifetime of their biosynthetic enzymes and corresponding mRNAs, although there is little information on this subject in the scientific literature. The lifespan of transcripts for SMs has been investigated in some Gram-positive bacteria [50,51,52]. In general, in eukaryotic cells, SM biosynthetic enzymes are degraded in proteosomes by proteases after ubiquitination, and in this way, the released amino acids are recycled.

Eisenberg et al. [53] reported that spermidine enhanced the longevity of yeasts, flies, worms, and animal cells; this was supported by the observation that ageing was coincident with a decrease in spermidine levels below critical concentrations in these distinct cells and resulted in programmed cell death. These authors also observed that administration of spermidine delayed cell death in ageing mice. This effect of spermidine was investigated with more detail in *S. cerevisiae* [54,55]. Addition of spermidine to yeast cultures resulted in histone H3 deacetylation due to inhibition of histone acetyl transferases in vivo and in vitro; conversely, lowering spermidine levels led to histone hyperacetylation, oxidative stress, and cell death [53]. Spermidine increases yeast life span, that is, the time that yeast cells remain viable, while delaying necrotic cell death. In yeasts, spermidine inhibits histone acetyl transferases such as iki3p and sas3p. Spermidine-treated yeast shows hypoacetylation at histone H3. This modification of the chromatin acetylation pattern leads to a reprograming of the transcriptome that affects genes related to peroxisomal degradation and formation of vesicle systems [54]. 

Spermidine provokes an important reorganization of the membrane systems in yeasts. In this reorganization process, the microtubule-associated protein LC3, also named ATG8 protein, is modified by lipidation, which favours its association with membrane systems [54]. Posttranslational modification of proteins by palmitoylation and other lipidations are linked to the formation of vesicles and secretory mechanisms in different filamentous fungi and related to the secretion of secondary metabolites (reviewed by [56]).

### An Increase in Vesicle Formation Is Correlated with Prolongation of the Half-Life of Transcripts of Penicillin Biosynthetic Genes

Prolongation of the half-life of penicillin transcripts follows the addition of spermidine to *P. chrysogenum* cultures [29]. This transcript life extension is in agreement with the increments in encoded enzyme levels, particularly of the third enzyme involved in penicillin biosynthesis, isopenicillin acyl transferase, that is encoded by the *penDE* gene [29]. This life extension of cultures supplemented with either spermidine or DAP is associated with the formation of numerous transport/secretion vesicles in *P. chrysogenum* [29]. Confocal microscopy indicates that the number of these vesicles increases rapidly at the end of the culture growth phase, coinciding with the stage of intense penicillin biosynthesis, and continues afterwards [29]. Microscopic analysis indicates that these vesicles contain microgranulated internal cargo material and are similar to the so-called multivesicular bodies observed in *P. chrysogenum* [57].

Similar detailed studies have been performed in *Aspergillus parasiticus* on the vesicles and vacuole systems involved in the biosynthesis and secretion of aflatoxins [58,59]. These authors purified a vacuole and transport vesicle fraction (V fraction) by sucrose gradient ultracentrifugation and stained it with fluorescent markers. This fraction was highly enriched in vacuoles and vesicles but was free of intact mitochondria and nuclei. The V fraction was re-purified by two rounds of sucrose ultracentrifugation and shown to contain mainly transport vesicles, endosomes, and vacuoles. LC/MS/MS analysis indicated that this fraction contained several enzymes involved in aflatoxin biosynthesis, including enzymes of the early, middle, and late pathway steps [58,60]. These data confirm that many of the membrane-bound subcellular compartments in the V fraction are vesicles which translocate a complex mixture of enzymes involved in primary and secondary metabolism to their destination within the cell or to the cell membrane for secretion. The enzyme activity of the V fraction has been demonstrated by in vivo addition of the intermediate versicolorin A, which is efficiently converted to aflatoxin [58,59]. Importantly, the V fraction also contains polyamines and polyamine biosynthetic enzymes, particularly the highly conserved spermidine synthase, an enzyme that converts putrescine to spermidine (Figure 1), supporting the important function of this polyamine in the control of the biosynthesis of aflatoxins and other SMs. Noteworthy, several enzymes involved in the biosynthesis of secondary metabolites other than aflatoxin were also located in the V fraction, e.g., the enzyme that synthesizes 6-methylsalicylic acid, an intermediate in the biosynthesis of patulin [59], suggesting that the V fraction is involved in the biosynthesis of several SMs and not only aflatoxins.

## 5. DAP and Spermidine Regulate the LaeA-Mediated Control of Secondary Metabolites

The *laeA* gene (so named for loss of *aflR* expression) was initially discovered in *A. nidulans*. LaeA is a nuclear protein with a methyltransferase domain [61] that appears to modify the heterochromatin organization. This protein is a member of the velvet protein complex formed by VeA, VelB, VelC, and VosA that coordinates the light or darkness signal with fungal development and secondary metabolite formation in *A. nidulans* [62].

### 5.1. DAP and Spermidine Positively Regulate Expression of the LaeA Gene in P. chrysogenum

In *P. chrysogenum* and *A. chrysogenum*, enhanced expression of *laeA* results in increased production of β-lactam antibiotics and other SMs [61,63].

The cloned *P. chrysogenum laeA*-gene-encoded protein is 61% identical to the homologous protein of *A. nidulans*; neither of these two fungi has the *laeA* gene linked to the penicillin gene cluster formed by the *pcbAB-pcbC* and *penDE* genes [63]. The *laeA* gene is present in a single copy in the *P. chrysogenum* genome, even in high-producing strains that contain the penicillin cluster amplified in tandem repeats [64]. Silencing of the *laeA* gene by antisense mRNA exerts a strong effect on penicillin biosynthesis, drastically reducing its production [63]. This result was confirmed using an *laeA* deleted mutant [65]. The *P. chrysogenum* LaeA protein has an S-adenosyl methionine binding site, as occurs with the protein of *A. nidulans*, and has been proposed to have methyltransferase activity, although the substrate of this methyltransferase activity is still unclear [63,66,67].

Also noteworthy is that DAP and spermidine revert the effect of the *laeA* knock-down mutation, suggesting that the effect of these diamines may be mediated by LaeA. The green colour of *P. chrysogenum* conidia and the brown mycelium pigment in solid medium cultures were lost in the *laeA* knock-down mutant, although these characteristics were partially regained by supplementation with DAP or spermidine [29]; in addition, the *laeA*-silenced mutant poor sporulation phenotype was also reverted after polyamine supplementation. Transcriptional studies of the *laeA* gene showed that it is poorly expressed in the growth phase of *P. chrysogenum* cultures, as occurs with other regulatory genes. RT-PCR studies showed that transcription of *laeA* in the wild-type strain was clearly enhanced by addition of DAP or spermidine, but not by putrescine, indicating that the effect was specific for the first two polyamines. This effect was particularly clear in the penicillin production of cultures of the knock-down *laeA* mutant, in which the residual production was enhanced 12- to 16-fold by addition of the polyamines, reaching levels above those of the wild-type strain [29,63]. Therefore, it was concluded that the DAP/spermidine-addition-mediated increase in *laeA* gene expression compensated the LaeA deficiency in the *laeA* knock-down mutant and complemented several physiological and morphological characteristics affected by LaeA in filamentous fungi.

### 5.2. DAP and Spermidine Simultaneously Regulate LaeA Formation and Secondary Metabolite Biosynthesis in Different Fungi

Spermidine and other polyamines are known to stimulate the production of different secondary metabolites, and this raises the question of whether all the effects of these polyamines on the biosynthesis of SMs are due to their action on *laeA* expression. LaeA control of secondary metabolites’ biosynthesis and the secretion of organic acid and extracellular enzymes has been studied in many filamentous fungi [42,66,68]. In *A. flavus*, supplementation of cultures with spermidine at concentrations as low as 0.5 mM increased 2.6-fold the expression of the *laeA* gene, simultaneously with the stimulation of aflatoxin biosynthesis. Supplementation with the tetraamine spermine has a very low effect, suggesting that the triamine spermidine is the most active polyamine in this regulatory mechanism [42].

As indicated before, in the plant pathogen *Fusarium graminearum*, the biosynthesis of trichothecenes is stimulated by diamines [41]; putrescine, the immediate precursor of spermidine, increases the yield of trichothecenes associated with increased expression of the trichothecene TRP5 and TRP6 genes and is probably mediated by LaeA.

In the lovastatin producer *A. terreus*, the polyamine effect on production of lovastatin is associated with the LaeA regulation of fungal metabolism. Expression of the *laeA* gene in polyamine-supplemented conditions was 1,2-fold in the wild-type strain and 1.6-fold in the high lovastatin producer as compared with non-supplemented ones. However, the possible connection of LaeA with the overexpression of the lovastatin biosynthetic genes was difficult to assess because of the large stimulation of production exerted by the specific *lovE* regulator that is present in two copies in the *A. terreus* HY strain. Both LovE and LaeA are positive regulators in the biosynthesis of lovastatin, but their effect is not additive [44].

## 6. DAP and Spermidine Induce the α-NAC Co-Activator

In the course of proteomic studies in *P. chrysogenum* [38], two overrepresented proteins were found in relation to the proteome of polyamine-unsupplemented cultures. These proteins play important roles in the structural and regulatory functions of the nascent polypeptide-associated complex (NAC). The first overrepresented protein (2.64-fold) after addition of either DAP or spermidine is a nascent polypeptide-associated complex, α-NAC chain, of 203 amino acids, which has been reported to function as a transcriptional co-activator in *S. cerevisiae* [69,70] and filamentous fungi [71,72]. The second protein overrepresented after DAP and spermidine addition is a probable suppressor of tom1, named Mpt4 in *S. cerevisiae*. This protein which appears as a duplicated spot with slightly different electrophoretic mobility, is overrepresented 4.3-fold. The protein is a ribosome-associated protein from *S. cerevisiae* required for optimal translation under nutrient stress [73].

The α-NAC co-activator was first studied in mice and yeasts. The NAC is well conserved in eukaryotes and even in archaea, and is associated with the nascent polypeptides as they leave the ribosomes protecting the nascent polypeptide of unappropriated interactions [74,75,76]. This complex consists of two subunits, α-NAC and β-NAC, both structurally similar, that form an α-β heterodimer; the components play different roles, including transcriptional control of the expression of genes involved in diverse physiological functions. In general terms, the NAC binds the polypeptides as they leave the ribosomes, but the α and β individual subunits have functions of their own. The α-NAC subunit contains a ubiquitin-associated domain, UBA, that is involved in the ubiquitination of proteins [77], and a protein fold that is required for the formation of the complex with its partner component [77]. The NAC is phosphorylated [78], and this results in its translocation into the nucleus [79].

### 6.1. The α-NAC Co-Activator in Mammals and Yeasts

In murine osteoblasts, the α-chain of the NAC interacts with the TATA-box binding sequence (TBP) of target genes, as shown by immunoprecipitation and protein binding [70]. Antibodies again the α-NAC subunit showed that this protein is localized both in the nucleus and the cytosol and has an RSEKKARK nuclear localization signal, NLS [70]. This nuclear targeting sequence is well conserved in the homologous proteins of *A. nidulans*, *P. chrysogenum*, and several other fungi (Table 1). The presence of NLS in the α-NAC components of different fungi supports the conclusion that this is a nuclear protein with a transcriptional co-activator function [79].

In *S. cerevisiae*, the two components of the NAC complex (named EDFG1 and EDFG2, for enhancer of Gal4p DNA binding) have been shown to have transcriptional activating activity. The α-NAC co-activator (EDFG2) is a protein of 174 amino acids and works as a heterodimer with the β-NAC subunit (EDFG1). These two components have similarity to the homologous proteins in mice and humans [70,80] and are encoded by separate genes in the yeast genome [70,81,82]. Both EDFG1 and EDFG2 contain an Nac domain that allows them to form dimers and, in addition, EDFG2 contains a ubiquitin binding domain in its C-terminal region, and their ubiquitination degree varies during the different yeast growth phases. Ubiquitination of the components is performed by the multicomponent complex CCR4-Not [83,84]. This complex is formed by nine subunits and plays a role in the control of many metabolic pathways conserved in eukaryotes. Particularly relevant is the targeting of the transcriptional factor TFIID to different promoters by this complex [85,86,87]. In summary, the function of the two components of NAC in *S. cerevisiae* is strictly controlled by proteins of the CCR4-Not complex, as occurs in other eukaryotic cells. Recently, in *S. cerevisiae*, a second β-NAC component (β-NAC2) was found which emerged by duplication of the β-NAC1 (EDFG1) and evolved rapidly [88].

Similarly, in the fission yeast *Schizosaccharomyces pombe*, the NAC is associated with ribosomes, but in a large part, it remains free in the cytosol [89]. The α subunit contains a ubiquitination domain UBA that is also present in the *S. cerevisiae* homologous subunit; however, in contrast to *S. cerevisiae*, the *Sch. pombe* mutants defective in the α subunit do not have any alteration in ubiquitin-mediated degradation. Mutants of *Sch. pombe* defective in the α-NAC component are resistant to the arginine analogue canavanine, suggesting that an increase in the arginine pool in the mutant provides more ornithine (see Figure 1), which is in agreement with the important role of polyamines in α-NAC-mediated gene expression.

### 6.2. The Transcriptional Co-Activator Function of the Nascent Polypeptide α-NAC Subunit in Different Filamentous Fungi

Since polyamines clearly enhance formation of the α-NAC co-activator subunit in *P. chrysogenum*, interesting questions are whether the same phenomenon occurs in different filamentous fungi and what is the function of this co-activator protein in those fungi is. In the last two decades, several articles have reported significant advances in the role of the α-NAC subunit in filamentous fungi, where it has a critical function in the control of growth, differentiation, pathogenicity, and production of SMs (Table 2).

The function of α-NAC in enhancing the expression of genes related to pathogenicity is really interesting, since it also affects plant protection against fungal infection [91]. During a fungal attack on a host plant, there is a complex biochemical interaction between the fungus and the plant. The fungus produces different substances, such as several SMs, proteins, lipids, and other compounds that allow recognition and favour penetration into plant cells, with the plant response triggering inducible defence mechanisms [94]. The grey fungus *Botrytis cinerea* is a pathogen of several plants, including grapes and tomato plants, in which it produces an important decrease in fruit production. In the search for determinants of resistance to infection by *B. cinerea*, a protein was found that produces resistance to fungal infection and draught tolerance in plants [71]. These authors, for the first time, purified to near homogeneity a protein that triggers disease resistance in tomato plants and several other effects, including draught tolerance. Using reverse genetics, three internal peptides of the purified protein were identified that allowed for establishing that the protein corresponds to the α-component of *B. cinerea* NAC [71]. This protein contains 212 amino acids and is 64% identical to the homologous *P. chrysogenum* protein [38].

**Figure 2:**
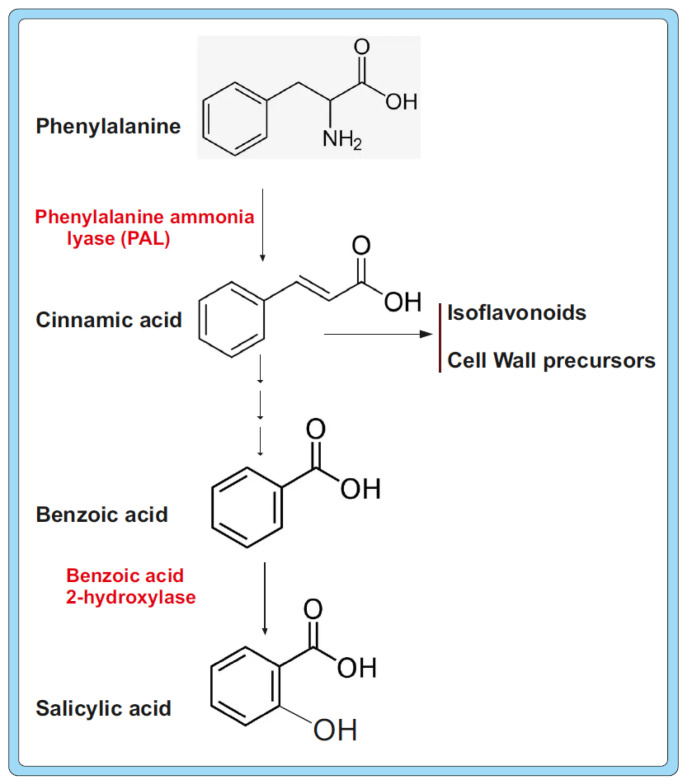
Biosynthesis of phenylalanine-derived isoflavonoids and salicylic acid. Note than the phenylalanine ammonia lyase (PAL) is the committing enzyme in the biosynthesis of these metabolites.

The purified protein stimulated germination and growth of wheat seeds when they were soaked in a solution of this protein and increased the draught tolerance of wheat plants after two cycles of draught stress. The mechanism of disease resistance triggered by the α-NAC subunit in *B. cinerea* is not yet fully elucidated; however, in tomato plants, an important hint is the observation that after supplementation with the α-NAC subunit, there was a 46% increase in the phenylalanine ammonium lyase (PAL) activity, an enzyme that is involved in the biosynthesis of phenylpropanoid-derived flavones, isoflavonoids, and lignin precursors [95] (Figure 2). In addition, peroxides and polyphenol oxidase activity increased by more than 100% in tomato plants supplemented with the α-NAC protein; these products and enzymes are involved in defence against *B. cinerea* infections.

Following studies of the effect of the α-NAC component in *B. cinerea*, other investigators searched for the implication of this protein for the development and pathogenicity of several filamentous fungi (Table 2). Li et al. [72] characterized the α-NAC subunit of *Sclerotinia sclerotiorum*, a necrotrophic plant pathogenic fungus that infects hundreds of plant species. This fungus forms sclerotia, a highly melanized structure that plays a key role in fungal dispersion and survival [96,97]. The α-NAC gene of *S. sclerotiorum* encodes a protein of 214 amino acids highly similar to those of *B. cinerea* and *P. chrysogenum*. Expression of the α-NAC encoding gene increases during the middle and late stages of sclerotia formation. Silencing of the α-NAC gene by antisense RNA results in less than 15% expression of α-NAC in relation to the parental strain. The α-NAC-silenced strain showed a delay in the maturation of sclerotia and a strong increase in the activity of pectin-degrading enzymes and polygalacturonidase gene expression, suggesting that α-NAC has a negative effect on fungal attack and virulence [73]. α-NAC also affected expression of the mitogen-activated protein kinase SK1, an enzyme known to be involved in the control of sclerotia formation [98]. The formation of sclerotia in filamentous fungi is closely associated with the biosynthesis of different secondary metabolites [99]. However, no further insight into the molecular mechanism that relates secondary metabolite biosynthesis and *S. sclerotiorum* pathogenicity has been reported, and additional research in this field is required.

The effect of α-NAC on plant pathogenic fungi was confirmed and extended by the role of α-NAC supplementation in different aspects of the metabolism of *Alternaria alternata* [90]. Pathogenic variants of *Alternaria* infect tangerines, lemons, apples, strawberries, and tomato plants, producing specific toxins. These toxins have been well characterized, and their biosynthesis and subcellular location have been the subject of several studies [100,101,102]. The *A. alternata* α-NAC (named Nac1 in this fungus) is a protein of 208 amino acids similar to those of *B. cinerea* and *S. sclerotiorum* [90]. This protein contains the α-NAC domain characteristic of the members of this protein family and a C-terminal ubiquitination associated domain. Analysis of different *nac1* mutants with either gain or loss of enzyme activity revealed that Nac1 is required for the formation and germination of conidia but has a negative effect on hyphal branching and the production of cell-wall-degrading enzymes. The Nac1 protein confers cellular susceptibility to reactive oxygen species (ROS) [90]. An outstanding finding was the observation that α-NAC is required for biosynthesis of the siderophore dimethylcoprogen in *A. alternata*. Distinct types of siderophores are known to be synthesized and secreted in filamentous fungi (reviewed by [103,104]). Wang et al. [90] confirmed previous evidence indicating that an NRPS (named NP6) is involved in the biosynthesis of dimethylcoprogen in *A. alternata* [105]. Siderophore-mediated iron acquisition plays a critical role in ROS detoxification [106]. Nac1 mutants are unable to detoxify ROS satisfactorily, in agreement with the essential role of siderophores in different physiological processes. The expression of Nac1 is positively regulated by iron, by the Hog1 mitogen activating protein kinase, and by the NADPH-dependent oxidase complex [107]. These authors suggested that a small increase in H_2_O_2_ induces the synthesis of dimethylcoprogen. In summary, these studies reveal that the α-NAC component is required for conidia development, pathogenicity, resistance to different osmotic stress factors, and siderophore biosynthesis for ROS detoxification; however, it is unclear whether α-NAC regulation of siderophore biosynthesis is common to other fungi or only to some genera, which requires further investigation. The purified *A. alternata* α-NAC injected in cucumber leaves induced plant defence reactions and decreased the infection caused by *Colletotrichum orbiculare* [91].

During a search of elicitors that protect plants against different pathogen attacks, a protein named PeaT1 was isolated from *Alternaria tenuissima* by Zhang et al. [92]. PeaT1 confers systemic resistance to tomato plants. This protein was recently identified as a member of the α-NAC co-activators family [92]. It consists of 207 amino acids and has 71 and 72% identity to the α-NAC protein of *S. sclerotiorum* and *B. cinerea*. The protein, purified after expression in *E. coli*, confers resistance to tobacco mosaic virus (TMV). Quantification of TMV revealed that there was a reduction in virus particle number and lesion size in the plant. PeaT1 induces two genes, which are markers for the systemic acquired resistance. Both genes are related to salicylic acid biosynthesis, as follows: (1) NPR1, which encodes the signal of the salicylic acid transduction pathway, and (2) the *pal* gene for the biosynthesis of salicylic acid itself [92,108]. Salicylic acid is synthesized from cinnamic acid, which is formed from phenylalanine by deamination by the PAL enzyme, then the side chain is shortened to benzoic acid and finally hydroxylation to form salicylic acid (Figure 2) [109]. Since the 1990s, it has been known that salicylic acid is essential in plants for the development of systemic acquired resistance.

## 7. The Metabolic Switch from Growth to Secondary Metabolites’ Production Phase Is Connected to Histone Modifications

**Figure 3:**
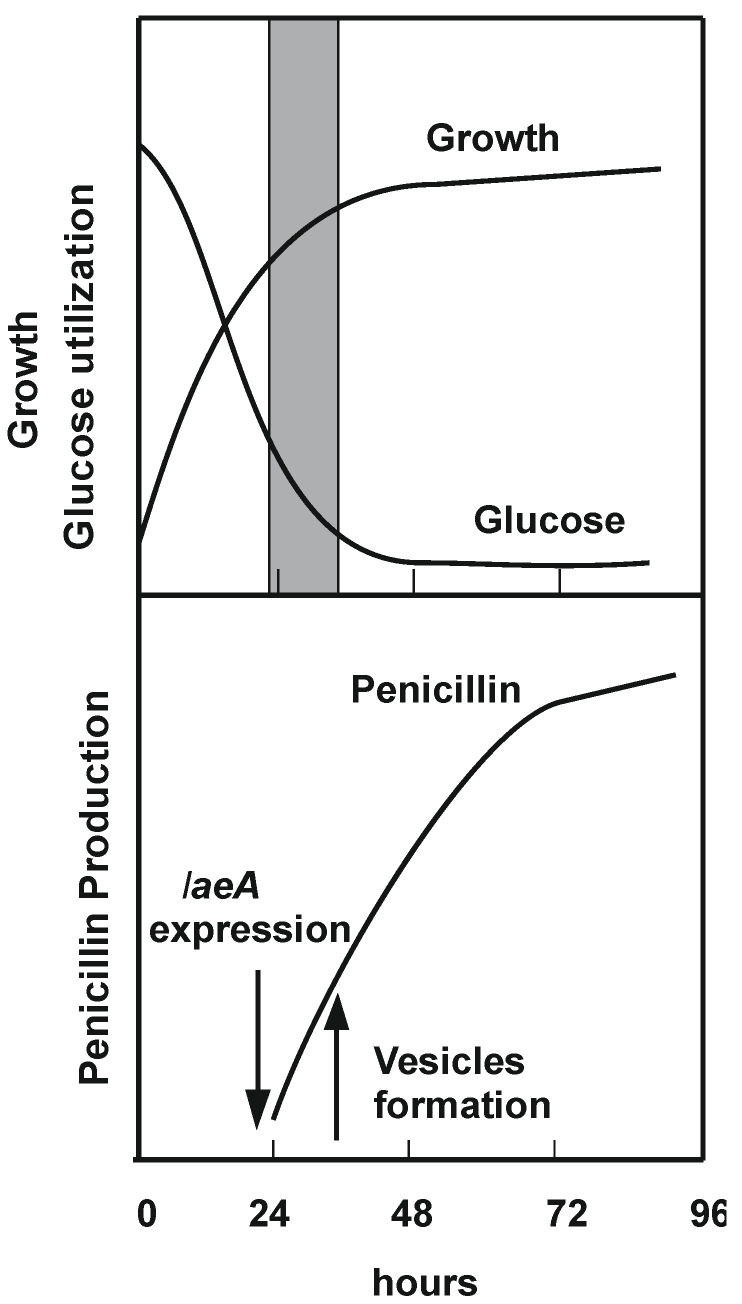
Scheme showing a model of the growth, transition phase, and production phase in *P. chrysogenum*. The model shows the time course of a limiting nutrient (glucose) culture, the onset of antibiotic production (penicillin), and the time of formation of LaeA and vesicles containing polyamines and spermidine synthase. The transition phase is shown with a grey column.

Most SMs are produced when the rapid growth phase of fungi declines because of the limitation of a key nutrient. An important metabolic switch (transition phase) was observed in *A. parasiticus* cultures in aflatoxin-producing medium, resulting in a burst of the formation of vesicles and the synthesis of aflatoxins biosynthetic enzymes and final products. This switch is not observed in non-permissive culture media [110]. These types of metabolic switches that trigger the conversion of a growth phase into an SM-producing stage are found in many fungal secondary metabolite production cultures (Figure 3), but the mechanism that induces the metabolic switch is still poorly known. Insight into the mechanism that induces this metabolic switch was reported in *A. parasiticus* when the sequential transcription initiation of aflatoxin early biosynthetic genes was correlated with the acetylation of histone H4, as shown by chromatin immunoprecipitation [111]. The role of H4p acetyl transferase in the metabolic switch of *Aspergillus* during the production of aflatoxin is highlighted by the finding that the cAMP receptor element (CRE1) binding protein (CRE1-BP) recruits histone acetyl transferases [111,112]. These authors propose that following nutrient limitation, CRE1-BP stimulates transcription by recruiting AflR and the histone acetyl transferase at the CRE1-regulated promoters. Aflatoxin biosynthesis is regulated by several mechanisms, of which the best known is activation by AflR, a binuclear zinc finger transcriptional factor. AflR recognizes sequences in most of the aflatoxin biosynthetic genes [113] and binds the DNA consensus sequence YCGNNNNNCGA; interestingly, there is a protein–protein interaction between AflR and the CRE1-BP, which, in turn, recruit histone acetyl transferases. CRE elements able to link the binding protein were found in several promoters of the aflatoxin gene cluster, e.g., in the *nor* gene that encodes the norsolorinic acid synthase [114]. Further progress in the characterization of the molecular mechanism of action of epigenetic histone modifiers will benefit from the use of novel advanced techniques such as RNAseq, which allows for genome-wide analysis of gene expression.

## 8. Heterochromatic Markers Are Associated with Repression of Secondary Metabolism Biosynthesis Genes

During the rapid growth phase of filamentous fungi, the heterochromatin structure is maintained by several modifications that avoid the expression of secondary metabolite biosynthetic genes, e.g., sterigmatocystin, terraquinone, and penicillin. The heterochromatin structure is characterized by high levels of heterochromatin 1 associated protein (HPA1) and by the presence of histone3-lysine9 trimethylated (H3K9). When some nutrients in the culture (e.g., phosphate, glucose, or an easily utilized nitrogen source) become limited, the HPA1 content of the heterochromatin decreases substantially, simultaneously with a decrease in the trimethylated H3K9 histone marker, and this results in a switch from growth metabolism to secondary metabolism production. Importantly, the global regulator LaeA counteracts markers that are associated with a lack of expression of secondary metabolism genes in heterochromatin, favouring the switch to euchromatin within the limits of the gene cluster region [25,115]. The metabolic switch is also concomitant with an increased level of acetylated H3 histone, an important marker associated with the expression of secondary metabolites genes [93].

The switch from primary to secondary metabolism that activates the expression of secondary metabolite biosynthetic genes is triggered by small molecules [116,117,118], including epigenetic modifiers [119,120]. The effect of small molecules as inhibitors of histone deacetylases has been supported by further research, in which the histone deacetylase encoding gene has been inactivated or deleted in fungi [121]. Moreover, modulation of the histone acetyltransferase and the switch in metabolism for the production of SMs have also been reported in other filamentous fungi by characterization of mutants altered or defective in the histone acetyltransferase gene [122]. This approach has been applied to the production of SMs by endophytic fungi [123]. Also noteworthy is that the biosynthesis of some SMs is activated in histone acetyl transferase-defective mutants, whereas the formation of other natural products is repressed [121], as also occurs in mutants defective in LaeA.

## 9. Conclusions and Future Outlook

The function of polyamines in the control of fungal metabolism has remained unclear for many years due to the multiple effects of these compounds. Polyamines are essential for cell growth and differentiation, but their role in fungal biosynthetic pathways remains enigmatic [30]. It is well known that they interact with chromatin, regulating its structure, mainly by influencing the condensation degree of nucleosomes. A low degree of condensation of the chromatin structure is critical for expression of the biosynthetic gene clusters that encode enzymes for the formation of a variety of SMs in fungi. A high degree of chromatin condensation is directly correlated with a low degree of histone acetylation. As described in this article, the effect of polyamines on gene expression is mediated by (1) the global regulator LaeA, a putative methyltransferase, although the substrate of methylation is unknown, and (2) by the α-NAC component of the nascent polypeptide-associated complex.

Scientific contributions in the last two decades have demonstrated that polyamines’ effect on the biosynthesis of penicillin, cephalosporin C, lovastatin, and aflatoxins is mediated by LaeA. Indeed, LaeA is a global regulator that controls the expression of hundreds of secondary metabolites in filamentous fungi [62,66,124]. These genes encode biosynthetic enzymes belonging to different types of fungal metabolites, including polyketides, non-ribosomal peptides, and isoprenoids. Polyamines induce expression of the *laeA* gene at the transcriptional level, and LaeA, in turn, affects the expression of many SMs.

In addition, it has been reported that DAP and spermidine increase the level of the α-NAC co-activator in *P. chrysogenum*, and this is supported by elucidation of the effect of α-NAC on the metabolism of several plant pathogenic fungi [72,73,90,92]. This co-activator recruits components of the transcriptional complex to the promoters of different fungal genes that, together with histone acetyltransferases, modify the expression of those genes. An important finding is the observation that the α-NAC co-activator is located in the nucleus and in the cytosol. Transport of the α-NAC into the nucleus is mediated by a nuclear localization signal (NLS) that has been found in the α-NAC proteins of many different fungi (Table 1) but is slightly different in the yeasts *S. cerevisiae* and *Sch. pombe*. The molecular mechanisms that control the transfer of the α-NAC protein into the nucleus may require phosphorylation [78]; this is an interesting subject for future research in this field.

The role of the α-NAC co-activator has been studied in several plant pathogenic fungi, e.g., the wheat and tomato pathogen *B. cinerea*. Research on resistance elicitors in this fungus resulted in the isolation of a protein that influences disease resistance and draught tolerance in the infected plant; by reverse genetics, this protein was identified as the α-NAC co-activator of this fungus [71]. Treatment of tomato plants with the purified α-NAC protein of *B. cinerea* induced the plant response by increasing the phenylalanine ammonia lyase levels, an enzyme involved in the formation of isoflavonoids and lignin precursors. However, more information on the molecular basis of the interaction of small metabolites produced by the infecting fungus with the plant defence mechanisms is needed. Mutants silenced in the expression of the α-NAC encoding gene in *S. sclerotiorum* showed delayed formation and maturation of sclerotia, a melanized structure associated with the formation of numerous SMs [99], therefore significantly affecting the biology of this pathogenic fungus. Sclerotia accumulates an impressive variety of SMs, including insecticides such as tetramic acid, indolditerpenoids, and diketopiperazines in *Aspergillus* species and ergot alkaloids in *Claviceps purpurea*. The presence of these SMs has been proposed to serve as a deterrent for the predation of fungi by insects. α-NAC also affects the biosynthesis of extracellular pectinolytic enzymes and expression of polygalacturonidase genes, which are required for the progress of fungal infection. The correlation of the biosynthesis of SMs in *S. sclerotiorum* and their role in pathogenicity is an interesting subject for future research. Studies on mutants of *A. alternata* deleted in the α-NAC gene stablished that this gene is required for the growth and formation/germination of conidia, although it has a negative effect on mycelium branching and the production of cell wall hydrolytic enzymes [90]. Most interesting was that α-NAC is required to synthesize the siderophore dimethylcoprogen, since it represses the formation of NP6, an NRPS required for its biosynthesis [104]. This finding is important, since it is known that siderophore-mediated iron acquisition and transport is critical for reactive oxygen species (ROS) detoxification [125]. α-NAC mutants are known to be deficient in the detoxification of ROS; this observation highlights the importance of the α-NAC protein in different fungal biological processes. It still remains to be elucidated whether this mechanism discovered in *A. alternata* is common to other filamentous fungi.

LaeA-mediated regulation of secondary metabolism biosynthesis has little in common with the α-NAC regulation of secondary metabolism, except for the fact that both of them act at the transcriptional level, although the action of α-NAC is not strictly similar to that of LaeA or other inducers, but it works as activator of the RNApol II together with other protein factors.

The described advances in polyamine induction of both the global regulator LaeA and the α-NAC co-activator provide novel information that opens a new research field to understand the relationship between fungal pathogenesis and the biosynthesis of SMs. The α-NAC component of the nascent polypeptide-associated complex has been characterized in several organisms, including mammals, yeasts, filamentous fungi, and archaea; it is known that the α-NAC protein interacts with the TATA binding protein (TBP), as shown by immunoblotting and protein binding studies [70], but further studies are required, particularly in filamentous fungi, to provide further insight into the interaction between the α-NAC co-activator and the transcriptional machinery. Advances in knowledge of the mechanisms that govern the expression of genes involved in SM biosynthesis is of upmost interest both in medicine and agriculture. In medicine, discovering novel compounds with immunosuppressant, antitumour, antiviral, or antibiotic activities, among others, is one of the more important tasks to advance the treatment of different human diseases, as well as advances in understanding the role of different fungal secondary metabolites in the pathogenicity of diverse plants, including the development of systemic acquired resistance that may protect plants against fungal or viral infections.

## Figures and Tables

**Table 1:** Comparative alignment of the NLS sequences in the α-NAC component of mammals with those of yeasts and filamentous fungi ^1^.

Origin	Sequence	α-NAC a.a.	GeneBank Access
*Mus musculum*	^70^SRSEKKARK^78^	215	AAB80961
*Penicillium chrysogenum*	^45^SRGEKKARK52	203	CAP86656
*Aspergillus nidulans*	^46^SRNEKKARK54	203	Q5AYK0
*Sclerotinia sclerotiorum*	^52^SRNEKKARK^60^	214	A7EIZ1
*Alternaria tenuissima*	^48^SRNEKKARK56	207	ABM54184
*Botrytis cinerea*	^51^SRNEKKARK^59^	212	A6SB28
*Saccharomyces cerevisiae*	^14^NKNEKKARE^22^	174	P38879

^1^ The amino acids that change in relation to the standard sequence of *Mus musculum* are shown in red color.

**Table 2:** α-NAC transcriptional co-activators in different yeasts and filamentous fungi.

Fungi/Yeasts	Amino Acids Number	Characteristics	References
*Saccharomyces cerevisiae*	174	Ubiquitin binding domain (UBA) in the C-terminal region	[81,82,88]
*Schizosaccharomyces pombe*	174	UBA in the C-terminal region. α-NAC mutants are resistant to the arginine analogue canavanine	[89]
*Penicillium chrysogenum*	203	Nac motif that allows formation of the α-β heterodimer. UBA at the C-terminal end	[38], This article
*Botrytis cinerea*	212	Triggers germination of wheat seeds and growth of the germline Protects wheat against draught Increases drastically the phenylalanine ammonia lyase (PAL) activity involved in flavin and isoflavonoids biosynthesis and in lignin precursor formation	[71]
*Sclerotinia sclerotiorum*	214	α-NAC silencing results in slow maturation of sclerotia and increases plant-cell-wall-degrading enzymes	[72]
*Alternaria alternata*	208	Contains the UBA and α-Nac domains α-NAC is required for biosynthesis of the dimethylcoprogen siderophore, synthesized by the NRPS np6 Siderophore-mediated iron acquisition plays an important role in ROS formation. α-NAC mutants are unable to detoxify ROS α-NAC is required for conidia development, plant pathogenicity, and resistance to osmotic stress factors	[90,91]
*Alternaria tenuissima*	207	Confers systemic acquired resistance (SAR) in infected plants ^1^ Protect tobacco plants against tobacco mosaic virus (TMV)	[92,93]

^1^ Triggers expression of two genes involved in SAR, one of them is an inducer of SAR and the second is the PAL for cinnamic acid biosynthesis that is converted to the salicylic acid signal.

## Data Availability

No new data were created or analyzed in this study. Data sharing is not applicable to this article.

## Abbreviations

The following abbreviations are used in this manuscript:

AflR	aflatoxins regulatory protein
cAMP	cyclic AMP
CoA	coenzyme A
CPC	cephalosporin C
CRE	cAMP receptor element
DAC	Deacetylcephalosporin C
DAP	1,3-diaminopropane
dcSAM	decarboxyl-SAM
DON	deoxynivalenol
EDFG2	α-NAC component in *S. cerevisiae*
EDFG1	β-NAC component in *S. cerevisiae*
H3K9	histone3 lysine9
HAT	histone acetyltransferase
HDAC	histone deacetylase
HPA1	Heterochromatin-associated protein 1
HY	high-yield-producing strain
*laeA*	Mutant producing “loss of *aflR* expression”
IAT	isopenicillin *N*-acyltransferase
NAC	nascent polypeptide-associated complex
NLS	nuclear localization signal
NMR	nuclear magnetic resonance
NRPS	Non-ribosomal peptide synthetase
PAL	phenylalanine ammonia lyase
PKS	polyketide synthase
SAM	S-adenosylmethionine
SAR	system-acquired resistance
SMs	secondary metabolites
TBP	TATA-box binding protein
TMV	tobacco mosaic virus
UBA	ubiquitin-associated domain
